# Genome-wide methylation and gene-expression analyses in thalassemia

**DOI:** 10.18632/aging.206037

**Published:** 2024-08-09

**Authors:** Wei Zhang, Xiaokang Li, Uet Yu, Xin Huang, Hongmei Wang, Yi Lu, Sixi Liu, Jian Zhang

**Affiliations:** 1 School of Medicine, Southern University of Science and Technology, Shenzhen 518055, Guangdong, China; 2 Shenzhen Key Laboratory of Cardiovascular Health and Precision Medicine, School of Public Health and Emergency Management, Southern University of Science and Technology, Shenzhen 518055, Guangdong, China; 3 Center for Reproductive Medicine, University of Hongkong-Shenzhen Hospital, Shenzhen 518053, Guangdong, China; 4 Department of Hematology and Oncology, Shenzhen Children’s Hospital, Shenzhen 518038, Guangdong, China; 5 Department of Infectious Diseases, Shenzhen Children’s Hospital, Shenzhen 518038, Guangdong, China

**Keywords:** thalassemia, blood, WGBS, RNA-seq

## Abstract

Thalassemia is the most common autosomal genetic disorder in humans. The pathogenesis of thalassemia is principally due to the deletion or mutation of globin genes that then leads to disorders in globin-chain synthesis, and its predominant clinical manifestations include chronic forms of hemolytic anemia. However, research on the epigenetics and underlying pathogenesis of thalassemia is in its nascency and not yet been systematically realized. In this study, we compared the results of RNA-seq and the whole-genome bisulfite sequencing (WGBS) on 22 peripheral blood samples from 14 thalassemic patients and eight healthy individuals revealed a genome-wide methylation landscape of differentially methylated regions (DMRs). And functional-enrichment analysis revealed the enriched biological pathways with respect to the differentially expressed genes (DEGs) and differentially methylated genes (DMGs) to include hematopoietic lineage, glucose metabolism, and ribosome. To further analyze the interaction between the transcriptome and methylome, we implemented a comprehensive analysis of overlaps between DEGs and DMGs, and observed that biological processes significantly enriched the immune-related genes (i.e., our hypermethylated and down-regulated gene group). Hypermethylated and hypomethylated regions of thalassemia-related genes exhibited different distribution patterns. We thus, further identified and validated thalassemia-associated DMGs and DEGs by multi-omics integrative analyses of DNA methylation and transcriptomics data, and provided a comprehensive genomic map of thalassemia that will facilitate the exploration of the epigenetics mechanisms and pathogenesis underlying thalassemia.

## INTRODUCTION

Thalassemia is a group of autosomally inherited hemolytic disorders and the most prevalent autosomal-recessive hemolytic disease worldwide [1], with approximately one to five percent of the global population estimated to be carriers of thalassemia [2–4]. Molecular biologic studies have revealed that thalassemia is caused by the deletion or mutation of the globin genes [5–7]. Adult hemoglobin is primarily composed of two α-globin subunits and two β-globin subunits [8–10]: the α-globin gene is located on chromosome 16, with each homologous chromosome containing two α-globin genes (i.e., *HBA1* and *HBA2*); and the β-globin gene is located on chromosome 11, with each homologous chromosome containing one β-globin gene (i.e., *HBB*). Thalassemia is thus principally segregated into two types in clinical practice, alpha thalassemia and beta thalassemia; although there exist a small number of unusual variants of thalassemia such as delta-beta thalassemia and delta thalassemia [9].

Conventional clinical treatments for thalassemia consist of blood transfusion, iron removal, and splenectomy. Allogeneic hematopoietic stem cell transplantation (HSCT) has more recently been shown to be the most effective therapeutic regimen to treat thalassemia [11–13]. Allogenic HSCT is limited to patients having human leukocyte antigen (HLA) homologous donors, and gene therapy using autologous hematopoietic stem cells provides an alternative treatment for allogenic HSCT [13, 14]. Although there are data supporting HSCT as relatively ideal in the setting of HLA matching, the probability of finding an appropriately histocompatible donor is less than 50% [15]. While previous studies showed no significant difference in the survival of patients receiving peripheral blood-derived stem cells and patients receiving bone marrow-derived stem cells, patients receiving the latter produced a faster engraftment rate and lower rejection rate than those patients receiving the peripheral stem cells [16]. Other studies encompassed peripheral blood, bone marrow, or umbilical cord blood from unrelated donors to adjust the myeloablative regimen and to reduce the toxic effects of the graft, thus significantly augmenting the overall survival rate [17, 18].

To reveal the complex pathogenesis of thalassemia, it is crucial to integrate multi-omics data (e.g., from genomics, epigenomics, proteomics, and metabolomics) to assess their relationships at different molecular levels and their impacts on disease phenotypes [19]. As an important component of epigenomics, DNA methylation is not only related to other epigenetic modifications, but also maintains an important relationship with gene expression [20–23]. Therefore, the integration of DNA methylation and gene expression and the systematic analysis of the relationship between the two comprise a currently exciting area of research. In this study we executed a comprehensive and systematic biologic analysis of 14 pediatric patients with thalassemia and eight age-matched healthy individuals by combining genome-wide transcriptional and global-DNA methylation analyses. By integrating epigenomic and transcriptomic data, we demonstrated an association between DNA methylation and differential gene-expression patterns in thalassemia, and explored the epigenetic mechanisms and pathogenesis underlying thalassemia.

## RESULTS

### Data from the research subjects

This study comprised a collection of peripheral blood samples from 14 pediatric patients with thalassemia (age range: 4-14 years, Mean±SD: 8±3.0) and eight healthy children (age range: 5-14 years, Mean±SD: 10±2.9) from which the RNA and DNA were sampled for transcriptomic and methylation sequencing, respectively. However, due to problems during the extraction processes in some research subjects, sample Th6 in the thalassemia group was only subjected to methylation sequencing, sample Th14 was only subjected to transcriptome sequencing, and in the control group sample N1 was only subjected to methylation sequencing. All of the remaining samples were subjected to both methylation and transcriptomic sequencing (see Table 1 for details).

**Table 1:** Table of sample characteristics.

**Samples**	**Gender**	**Age (years)**	**Type**	**Sequencing**
Th1	M	8	CD41-42/ CD41-42	WGBS/RNA-seq
Th2	F	10	41-42M/17M	WGBS/RNA-seq
Th3	F	8	CD41-42/IVS-2-654	WGBS/RNA-seq
Th4	M	14	CD41-42/ CD41-42	WGBS/RNA-seq
Th5	F	7	SEA	WGBS
Th6	F	13	CD41-42/IVS-I-654	WGBS/RNA-seq
Th7	F	5	IVS-II-654/βE	WGBS/RNA-seq
Th8	M	8	654M/17M	WGBS/RNA-seq
Th9	M	6	IVS-II-654/ IVS-II-654	WGBS/RNA-seq
Th10	F	11	CD41-42/βE	WGBS/RNA-seq
Th11	M	4	-SEA/ cs	WGBS/RNA-seq
Th12	F	7	CD41-42/IVS-2-	WGBS/RNA-seq
Th13	F	6	IVS-1-128/-28	WGBS/RNA-seq
Th14	F	5	CD41-42/IVS-1-1	RNA-seq
N1	M	12	Normal	WGBS
N2	F	11	Normal	WGBS/RNA-seq
N3	M	12	Normal	WGBS/RNA-seq
N4	F	7	Normal	WGBS/RNA-seq
N5	F	9	Normal	WGBS/RNA-seq
N6	M	14	Normal	WGBS/RNA-seq
N7	F	10	Normal	WGBS/RNA-seq
N8	M	5	Normal	WGBS/RNA-seq

### RNA-seq and WGBS quality-assessment and alignment summary

All samples in the present study generated high-quality RNA-seq reads, fastp pre-processing was used for raw-read quality, and we obtained over 99% clean data [24] (Supplementary Table 1). The HISAT2-alignment program for mapping sequencing reads was implemented to carry out comparative analysis based on the reference genome; the number of reads and the proportion of effective reads (i.e., the total mapped) for each sample were both ≥96% (Supplementary Table 2). We then calculated the distribution position of the reads in the reference genome according to all the reads that were mapped to the genome (i.e., the total mapped reads). Genome-wide DNA methylation analysis was consequently performed on the samples. In brief, the clean reads obtained from methylation sequencing were filtered to obtain high-quality clean reads for our subsequent data analysis. Each sample showed ≥97% high-quality clean reads (Supplementary Table 3).

### Cluster analysis

**Figure 1:**
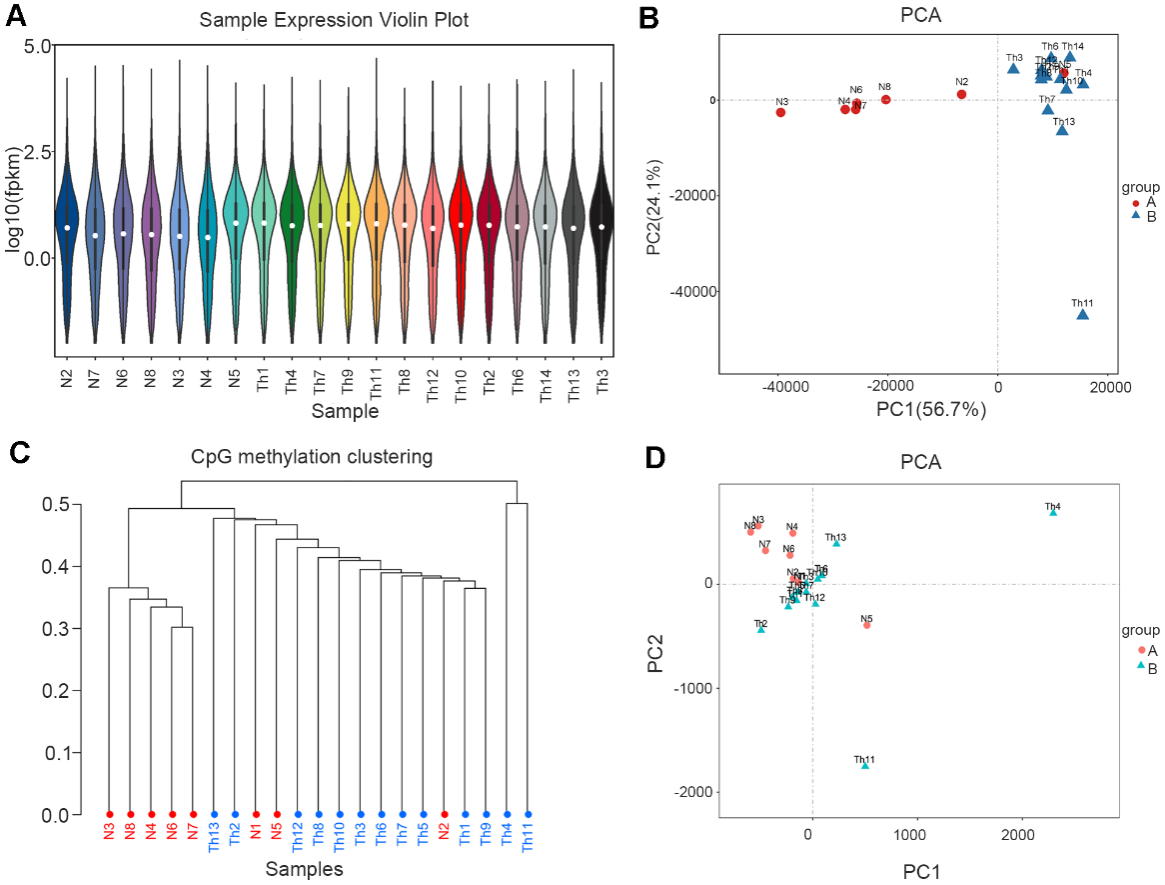
**RNA-seq and WGBS data cluster analysis.** (**A**) Sample expression violin plot. White dot represents the median Q2. The black rectangle represents the range from the lower quartile to the upper quartile. (**B**) Principal Component Analysis. Red dots represent normal. Blue dots represent thalassemia. (**C**) Principal Component Analysis (WGBS). Red dots represent normal. Blue dots represent thalassemia. (**D**) CpG methylation clustering. Red represents normal. Blue represents thalassemia.

The expression distribution of different sample genes or transcripts was displayed via the expression-distribution map and based on the fragments per kilobase of transcript per million mapped reads (FPKM) of each gene (Figure 1A). Dimensionality reduction was then used to ascertain the distance relationships between samples using principal component analysis (PCA). In the control group, samples N2 and N5 were outliers that were consistent with the thalassemic group, especially N5 was same with thalassemia group (Figure 1B). In methylation sequencing, the methylation-rate data for CpG sites in each sample were used to perform PCA and cluster analysis on all samples, and our results showed that three samples (N1, N5, and N2) were outliers in the control group (Figure 1C, 1D).

### Analysis of DEGs

**Figure 2:**
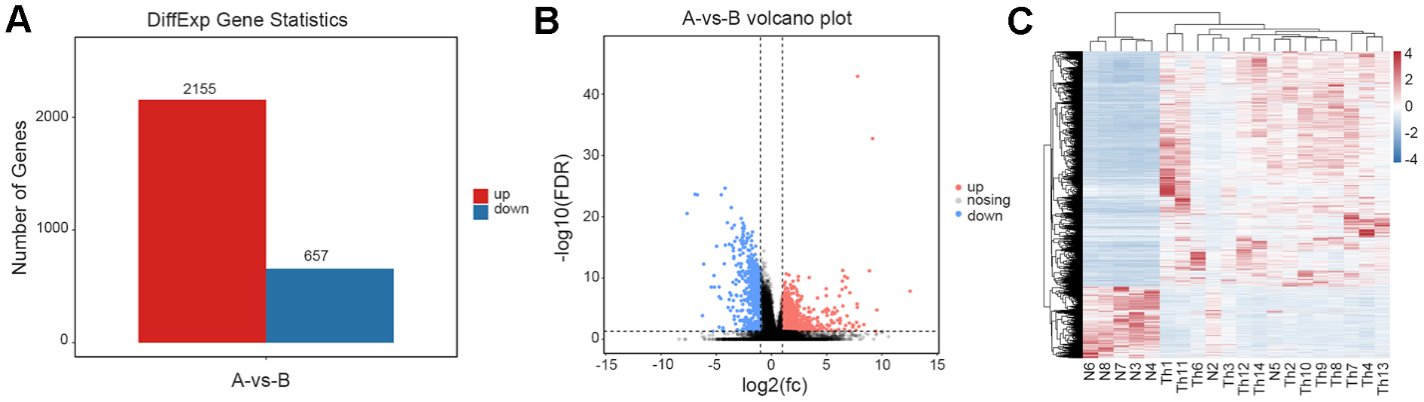
**Analysis of differentially expressed genes.** (**A**) The number of differentially expressed genes between normal and thalassemia group. Red represents up expression; Blue represents down expression. (**B**) The volcano plot shows the differentially expressed genes between normal and thalassemia group. Each dot corresponds to a gene. Red dots represent up expression; Gray dots represent not significant; Blue dots represent down expression. (**C**) Heatmap of differentially expressed genes in two groups. Red represents high expression; Blue represents low expression.

Via statistical analysis of the differences among genomes, genes with an FDR6 (six percent) <0.05 and |log2 fold-change| >1 were screened as significantly differentially expressed genes (DEGs); and in the thalassemic group, we uncovered 2,155 upregulated and 657 downregulated genes (Figure 2A). We generated volcano plots based on the DEGs in the comparison group (Figure 2B), executed hierarchical clustering of differential gene-expression patterns, and created a heatmap to present the clustering results (Figure 2C). Our results confirmed that samples N2 and N5 deviated from the control group.

### Functional-enrichment analysis of DEGs

**Figure 3:**
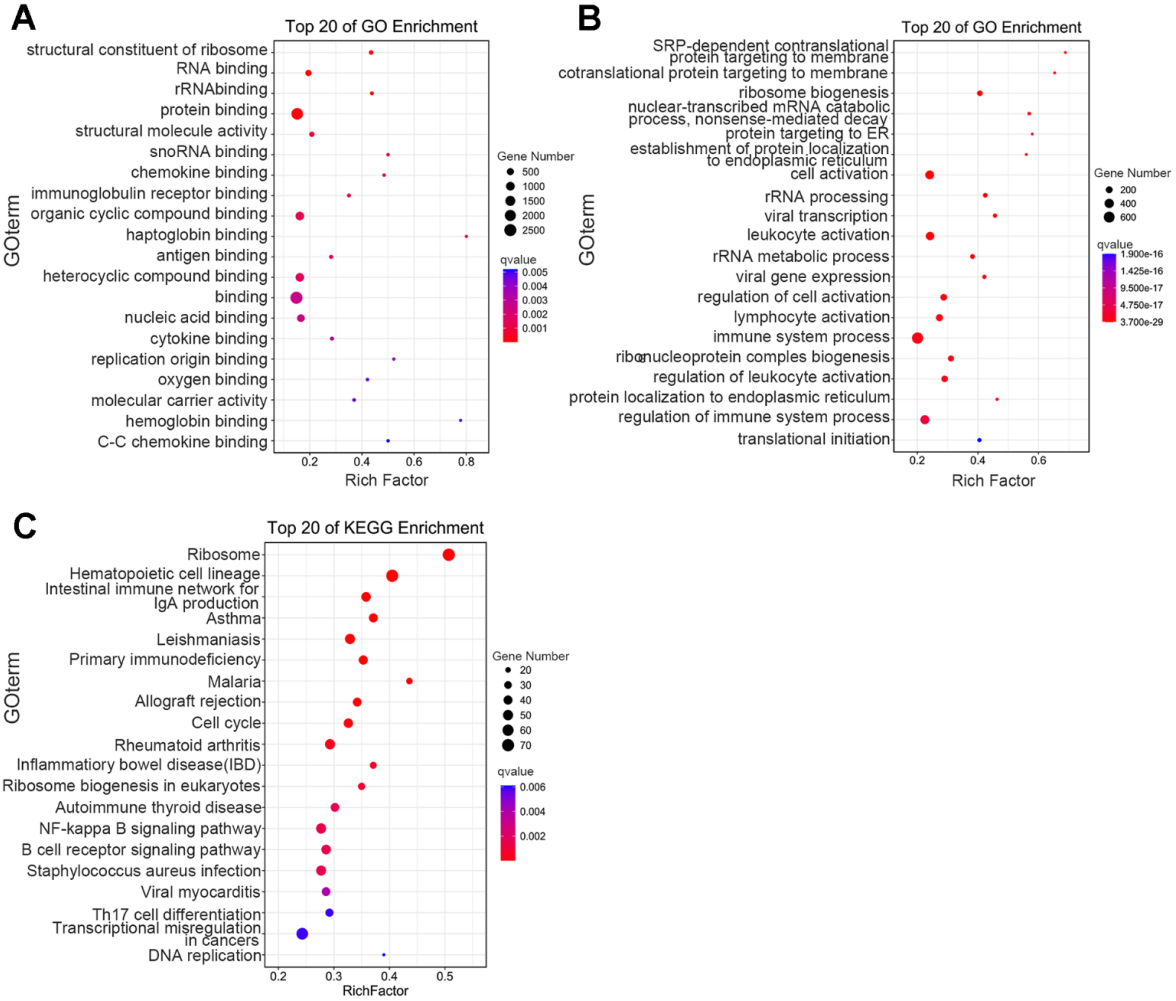
**Functional-enrichment analysis of differentially expressed genes by GO and KEGG.** The 20 most significantly enriched biological functions using GO (**A**, **B**) and KEGG (**C**) are illustrated in dot plots. Rich factor refers to the proportion of DEGs belonging to a specific term. Node size (gene number) refers to the number of DEGs within each term and node color indicates the level of significance (−log10 p-value).

To better understand the biologic functions related to DEGs, we executed Gene Ontology (GO)-enrichment analysis, and noted that molecular function was significantly enriched in haptoglobin binding, cytokine binding, structural constituent of ribosome, oxygen binding, and immunoglobulin receptor binding—indicating that in addition to the changes in globin binding, hemoglobin binding, and oxygen binding, many other related molecular functions were also significantly different in the thalassemia group (Figure 3A). We also found enriched biological processes such as immune system process, cell activation, ribosome biogenesis, leukocyte activation, and lymphocyte activation—indicating that the immune system of thalassemic patients was significantly different from that of healthy individuals (Figure 3B).

Significantly enriched pathways were established among DEGs through further GO- enrichment analysis, and our results showed that among the top 20 most significant differences, the second-highest ranking was reflected by hematopoietic cell lineage—indicating significant changes in the hematopoietic cell lineage-related genes in thalassemia patients (Figure 3C). We also observed significant changes in the expression of multiple transcription factors and epigenetic-modification enzymes in the DEGs, including DNA methyltransferases and deacetylases. Quantitative PCR was used to verify the results and to confirm that they were consistent with sequencing (Figure 3D).

### DNA-methylation changes

**Figure 4:**
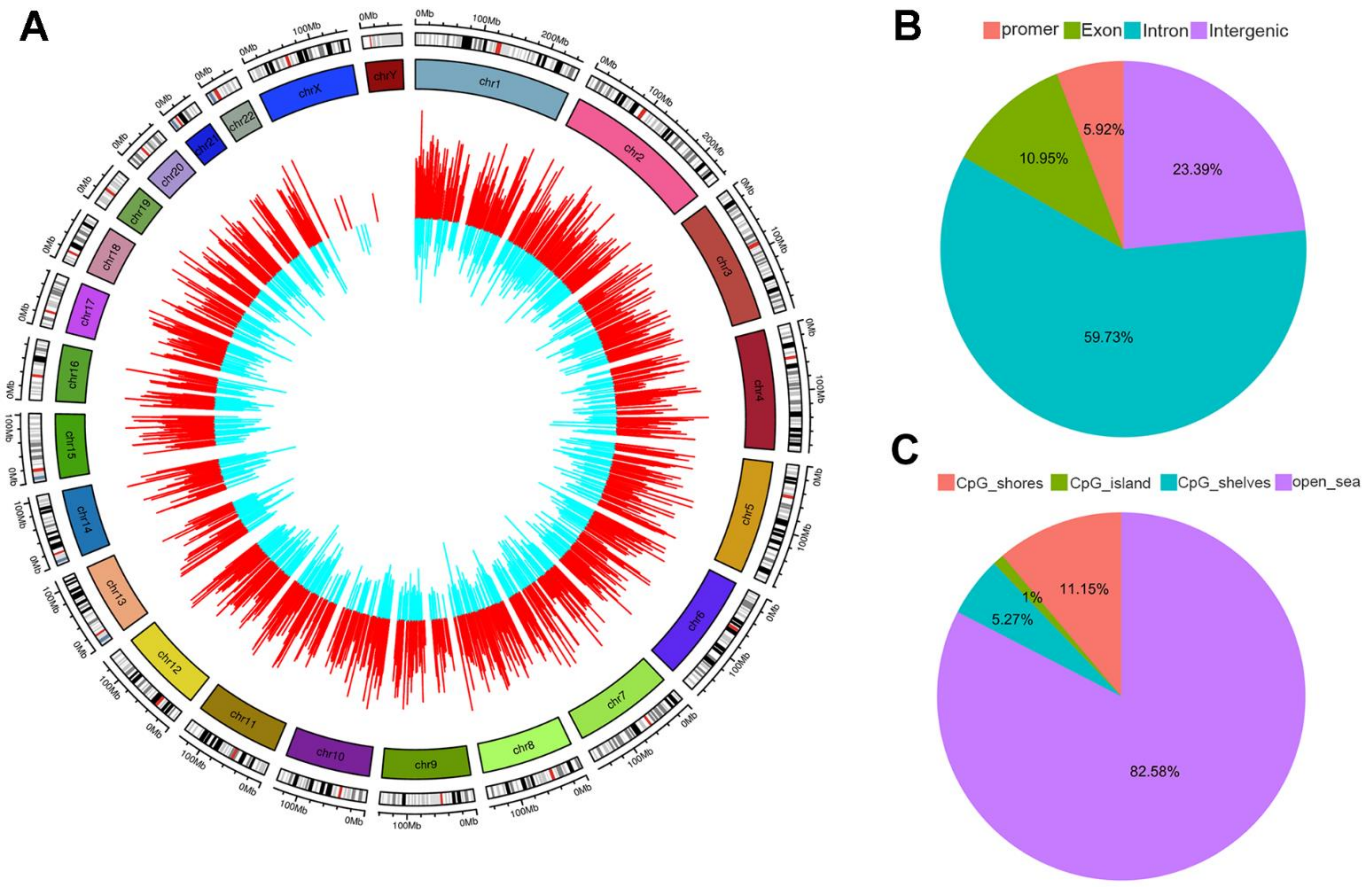
**Distribution of differentially methylated CpGs (DMR).** (**A**) Outer circle represents hypermethylated CpGs colored in red. Inner circle represents hypomethylated CpGs colored in blue. The height of each bar indicates the methylation change between thalassemia and normal. The distributions of DMR summarized based on genomic location (**B**) and relative to CpG islands (CpGi) (**C**).

Analysis of differentially methylated CpG sites (DMCs) revealed that there were 5,793 upregulated and 2,629 downregulated genes with regard to CpG site methylation in the thalassemia group (Data were not shown), indicating an overall effect of thalassemia on genome-wide methylation (Figure 4A). We also determined the genomic distribution of DNA methylation changes and the distribution of DMCs associated with CpG islands, and demonstrated that 5.92% of DMCs were located in promoter regions, 10.95% in exons, 23.39% in introns, and 59.73% of DMCs were located in intergenic regions (Figure 4B). In addition, most of the DMCs were located in “non-CpG-rich regions” (denoted as “open sea” in Figure 4C).

### Functional-enrichment analysis of differentially methylated genes (DMGs)

**Figure 5:**
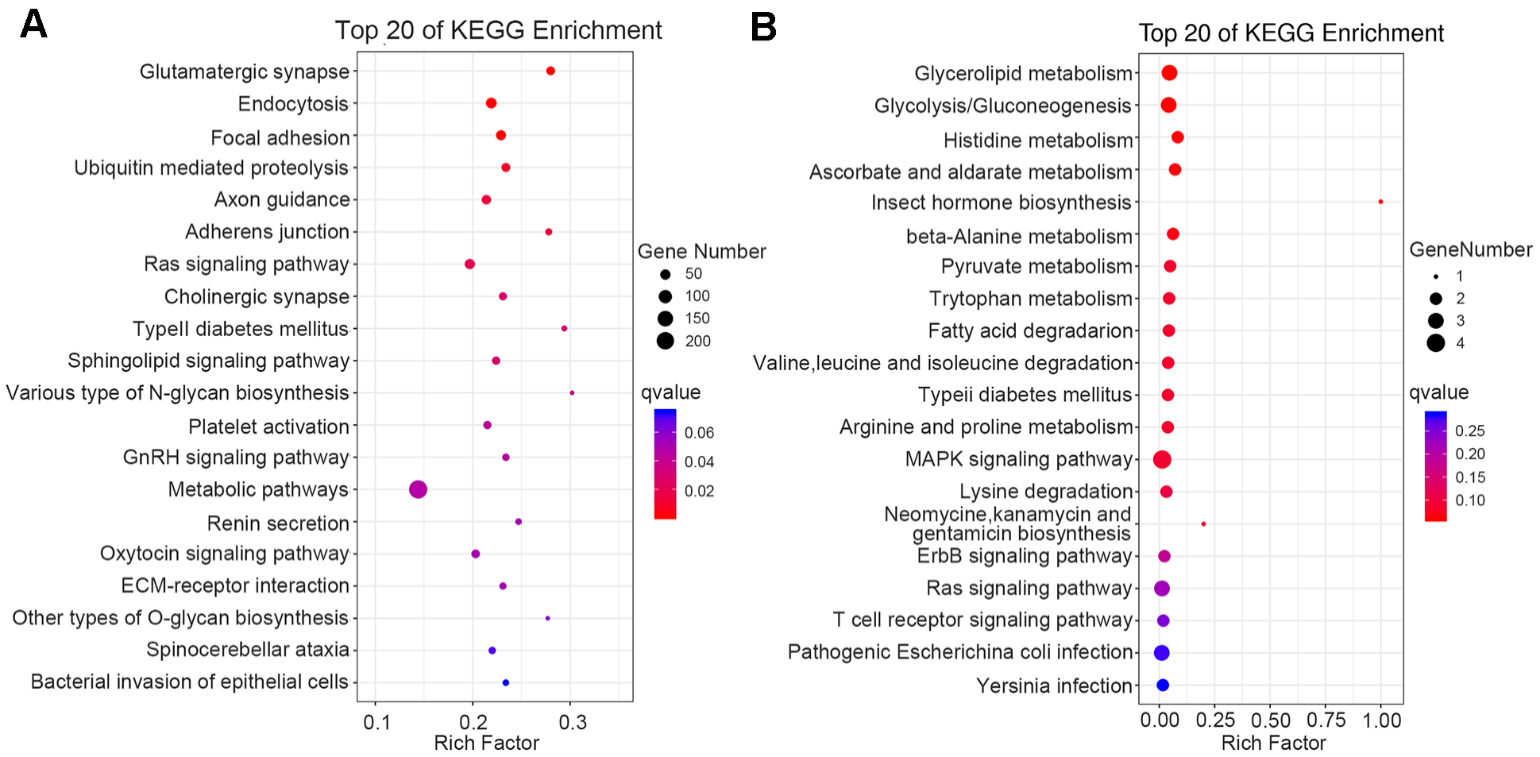
**Functional enrichment analysis of DMGs by KEGG.** The 20 most significantly enriched biological functions in DMC (**A**) and DMR (**B**) using KEGG are illustrated in dot plots. Rich factor refers to the proportion of DMGs belonging to a specific term. Node size (gene number) refers to the number of DMGs within each term and node color indicates the level of significance (−log10 p-value). DMGs mean differentially methylated genes.

We executed GO and Kyoto Encyclopedia of Genes and Genomes (KEGG)-enrichment analyses on the biological functions and signaling pathways of DMGs linked with thalassemia. Through KEGG-enrichment analysis of genes associated with the DMCs on the CpG sites, we obtained pathways of type II diabetes mellitus, platelet N1tivation, metabolic pathway, and ECM-receptor interaction (Figure 5A). Analysis of differentially methylated regions (DMRs) revealed that there were 82 upregulated genes and seven downregulated genes (Data were not shown). KEGG pathway-enrichment analysis identified glycolysis/gluconeogenesis, pyruvate metabolism, fatty acid degradation, type II diabetes mellitus, MAPK signaling pathway, and T cell receptor signaling pathway (Figure 5B).

### Analysis of overlap between DMGs and DEGs

**Figure 6:**
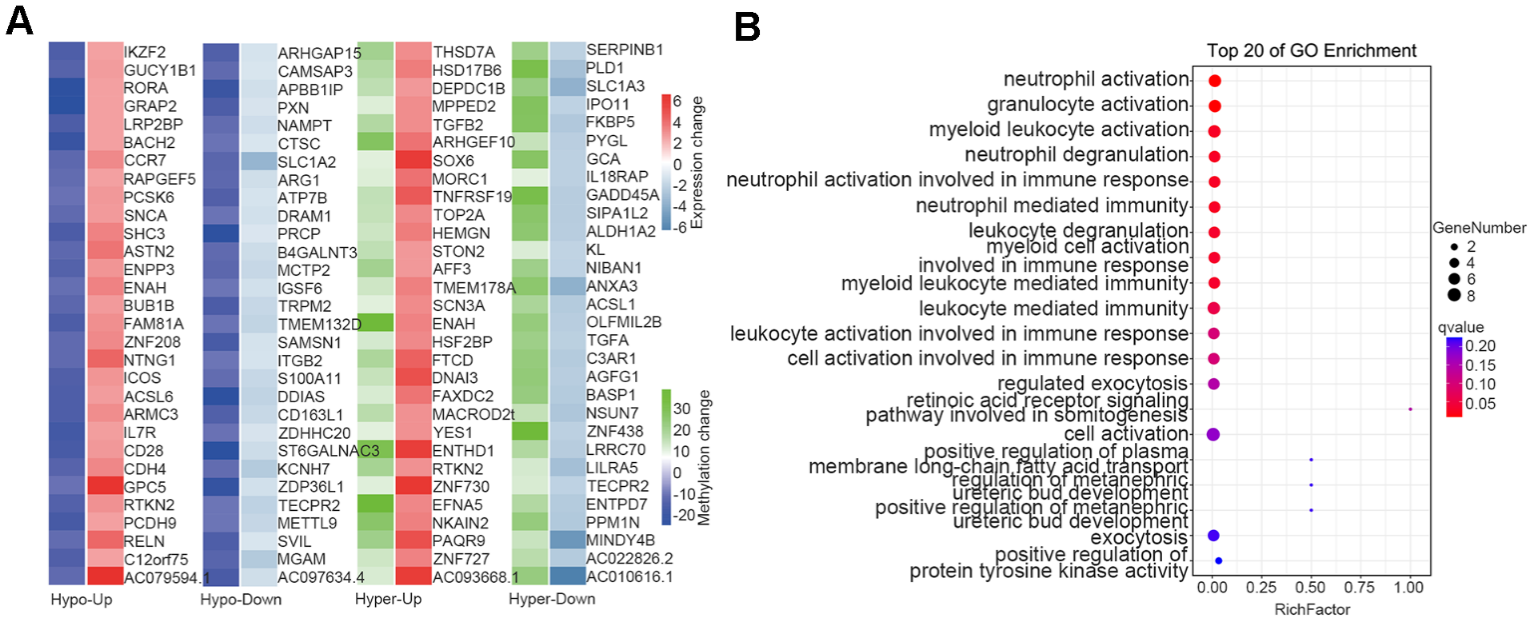
**Overlap between DMGs and DEGs.** (**A**) Heatmap of methylation and expression changes of the overlapping DMGs and DEGs. The first column of each group corresponds to the methylation change (Blue: Hypomethylated, Green: Hypermethylated), while the second column represents the gene expression change (Red: Upregulated, Right blue: downregulated). (**B**) Pathway enrichment analysis of overlapping genes in methylation and mRNA datasets. The 20 most significantly enriched pathways are illustrated in dot plots. Gene ratio refers to the proportion of DEGs belonging to a specific term. Node size (count) refers to the number of DEGs within each term and the color indicates the level of significantly (− log10 P-value). DMGs differentially methylated genes, DEGs differentially expressed genes.

To understand the relationship between epigenetic regulation and transcriptomic changes in the blood of thalassemic patients, we undertook an integrated analysis of DMGs and DEGs (after removing three outliers: samples N1, N5, and N2), and noted that the difference in the positional relationship between DMGs and coding genes affected the regulatory effect of DNA methylation. Figure 6A depicts the analysis of changes in DMG locations and DEG-expression levels, and further overlap analysis between the 2,323 DMGs and 4,442 DEGs showed overlap in 779 genes (Data were not shown). We divided these 779 genes into four categories N1cording to the direction of DNA methylation and gene expression: “Hypo-Down” (for hypomethylated and downregulated genes); “Hypo-Up” (for hypomethylated and upregulated genes); “Hyper-Down” (for hypermethylated and down-regulated genes); and “Hyper-Up” (for hyper-methylated and up-regulated genes). We then selected the genes with the top 30-fold differences in gene expression in eN1h category (Figure 6A), and GO-enrichment analysis indicated that immune-related genes were principally enriched in biological processes (i.e., our Hyper-Down group, Figure 6B).

## DISCUSSION

We herein collected peripheral blood samples from pediatric patients with thalassemia and performed genome-wide DNA methylation and transcriptomics analyses to elucidate the molecular changes occurring within the blood cells of thalassemic patients. Via the GO-enrichment analysis of DEGs, cell part, cell surface, and side of membrane were significantly enriched in the cellular component. Unbalanced globin-chain synthesis results in the precipitation of excess globin chains in erythrocyte precursors, and results in structural changes to the cell membrane. Athanasiou et al. used a micropipette aspiration technique to measure the ratio of the elastic-shear moduli of red blood cells in thalassemia-model mice and patients with thalassemia, and found that the rigidity of the thalassemic red blood cells was significantly greater than that of normal red blood cells [25]. A previous study showed that the reduction in erythrocyte deformability and membrane stability resulted in the destruction of erythrocytes as they passed through bone marrow-cavity blood vessels, the splenic sinus, and capillary networks—shortening their lifespan in the circulation [26]. We also performed KEGG-enrichment analysis, and noted that the second highest-ranked object of the top 20 most significant differences was hematopoietic lineage, indicating robust changes in hematopoietic lineage-related genes in thalassemic patients (Figure 3C). We additionally uncovered significant changes in the expression of multiple transcription-factor genes and epigenetic-modifying enzymes among the DEGs.

The KEGG-enrichment analysis of DMGs showed that type II diabetes mellitus and platelet activation were most enriched, and our analysis of DMRs showed that KEGG was enriched in glycolysis/gluconeogenesis, type II diabetes mellitus, and T cell receptor signaling pathway—among others (Figure 5B). Numerous studies have revealed that iron overload was correlated with diabetes. For example, Chen et al. showed that patients with type II diabetes manifested higher serum ferritin than healthy individuals [27] and Liang et al. demonstrated that children with high serum ferritin levels exhibited a higher prevalence of impaired fasting glucose than children with low levels [28]. Ansari et al. showed that nearly one-third of β-thalassemia patients who exhibited iron overload were insulin resistant, and that the insulin-resistance index increased with age and the elevation in serum ferritin, suggesting a close relationship between iron overload and islet resistance [29]. Luo et al., in a study of 79 patients with thalassemia, 114 patients with hemoglobin-H disease, and 18 patients with hemoglobin E-beta thalassemia, observed 33 cases of hypoglycemia, 25 cases of impaired glucose tolerance, and four cases of diabetes. In that study patients with thalassemia demonstrated symptoms that ranged from impaired glucose tolerance to symptomatic diabetes [30]. Although thalassemia patients who are dependent upon continual blood transfusions without adherence to iron chelators are at high risk of developing diabetes, there exists a broad therapeutic window that spans the spectrum from abnormal glucose metabolism to symptomatic diabetes. Intensive removal of iron thus reduces insulin resistance and is expected to delay the onset of diabetes.

With the development of biotechnology, gene insertion and gene editing have become strategies to correct and replace ineffective β globin in patients with β thalassemia [31–34]. Considerable efforts have also been made to study pharmacological drugs stimulating the production of γ globin and HbF [35, 36]. Allogeneic hematopoietic stem cell transplantation (HSCT) has been used successfully in the past few decades to provide curative treatment for transfusion-dependent patients, but only for a small number of patients with compatible donors [37, 38]. However, blood transfusion is more affordable as a traditional therapy. A wide spectrum of immune abnormalities has been described in numerous studies involving β-thalassemia patients with multiple transfusions—with iron overload a major cause of immunodeficiency in β-thalassemia. Since humans lack an efficient mechanism with which to excrete excess iron, chronic blood transfusions may lead to iron N1cumulation and thus generate reactive oxygen species that can trigger lipid, protein, DNA, and subcellular organellar damage, which can then cause cellular dysfunction, apoptosis, and necrosis, and precipitate target organ toxicity and dysfunction [39]. Our analysis of the overlap between DMGs and DEGs revealed that the immune-related genes (Hyper-Down group) were enriched in biological processes, indicating alterations to the immune system of patients with thalassemia relative to healthy individuals.

With the treatment of thalassemia has developed significantly, resulting in an increase in life expectancy. At the same time, a number of new onset and chronic diseases have emerged, including cancer. Over the years, several cases of solid and hematologic malignancies in patients with thalassemia have been reported in the literature [40–42]. In our study, KEGG results showed that in addition to immune-related diseases being enriched, cancer-related signaling pathways such as cell cycle, DNA replication, and NF-κB signaling pathways were also significantly enriched. Although the mechanisms underlying cancer development in patients with thalassemia are not well understood, studies have shown that patients with TDT and β thalassemia major have a higher risk of developing malignancies compared with the general population [43].

We herein analyzed the transcriptomes and methylomes of thalassemic patients to determine the relationship between epigenetics and gene-expression levels in thalassemia and uncovered enrichments in biological pathways such as hematopoietic lineage, immunity, glucose metabolism, and ribosomes. Furthermore, tumor-associated signaling pathways were enriched, such as NF-κB signaling pathway. Although the exact associations between these pathways and thalassemia remain unclear, they may be of great significance in understanding the molecular underpinnings of thalassemia, and should facilitate the discovery of novel genes related to its epigenetic regulation.

## MATERIALS AND METHODS

### Samples collection

This subject has been approved by the Medical Ethics Committee of Southern University of Science and Technology (Project number: SUSTC-JY2017041). The study was conducted in accordance with the ethical standards as laid down in the 2018NL-106-02 Declaration of Helsinki and its later amendments or comparable ethical standards. We collected peripheral blood samples from 14 pediatric patients with thalassemia (age range: 4-14 years, Mean±SD: 8±3.0) and 8 healthy children (age range: 5-14 years, Mean±SD: 10±2.9). Patient characteristics are given in Table 1. RNA and DNA were extracted for transcriptomic and methylation sequencing, respectively. However, sample Th6 and N1 were only subjected to methylation sequencing, and Th14 was only subjected to transcriptome sequencing.

### Genome-wide gene expression and methylation profiling

Total RNA Trizol reagent kit (Invitrogen, CA, USA) according to the manufacturer’s protocol. RNA quality was assessed on an Agilent 2100 Bioanalyzer (Agilent Technologies, CA, USA) and checked using RNase free agarose gel electrophoresis. Eukaryotic mRNA was enriched by Oligo(dT) beads, while prokaryotic mRNA was enriched by removing rRNA by Ribo-ZeroTM Magnetic Kit (Epicentre, WI, USA). Then the enriched mRNA was fragmented into short fragments and second-strand cDNA were synthesized by DNA polymerase I, RNase H, dNTP and buffer. QiaQuick PCR extraction kit (Qiagen, The Netherlands) was used to purify the cDNA fragments, and ligated to Illumina sequencing adapters. RNA-seq was used Illumina HiSeq2500 by Gene Denovo Biotechnology Co. (Guangzhou, China).

Genomic DNA (gDNA) was extracted from whole blood using DNeasy Blood & Tissue Kit (Qiagen, CA, USA) according to the manufacturer’s protocol. DNA concentration and integrity were detected by NanoPhotometer® spectrophotometer (Implen, CA, USA) and Agarose Gel Electrophoresis respectively. For library construction, genomic DNAs were fragmented into 100-300bp by Sonication (Covaris, Massachusetts, USA) and purified with MiniElute PCR Purification Kit (Qiagen, MD, USA). After purification, a single adenosine was added to the 3′ends of the fragmented DNA. And then adapter-ligated DNA fragments were treated. Fragment with adapters were bisulfite converted using Methylation-Gold kit (Zymo, CA, USA), unmethylated cytosine is converted to uracil during sodium bisulfite treatment. Finally, the converted DNA fragments were PCR amplified and sequenced using Illumina HiSeqTM 2500 by Gene Denovo Biotechnology Co. (Guangzhou, China).

### Data filtering

Reads obtained from the sequencing machines include raw reads containing adapters or low quality bases which will affect the following assembly and analysis. To get high quality clean reads, reads were further filtered by fastp (version 0.18.0) [24]. Briefly, we removed reads containing adapters, containing more than 10% of unknown nucleotides (N), and low quality reads containing more than 50% of low quality (Q-value ≤ 20) bases for RNA-seq. We removed reads containing more than 10% of unknown nucleotides (N), and low quality reads containing more than 40% of low quality (Q-value ≤ 20) bases for methylation profiling.

### Principal component analysis

R package Seurat v3.1.2 was used to process the single-cell data expression matrix. The data were first normalized by ‘NormalizeData’. ‘FindVariableGenes’ was then used to identify 2000 highly variable genes. Principal component analysis (PCA) was performed with R package gmodelsin this experience. PCA is a statistical procedure that converts hundreds of thousands of correlated variables (gene expression/methylation level) into a set of values of linearly uncorrelated variables called principal components. PCA is largely used to reveal the structure/relationship of the samples/datas.

### Differentially expressed genes (DEGs)

RNAs differential expression analysis was performed by DESeq2 software between two different groups [44]. The genes/transcripts with the parameter of false discovery rate (FDR) below 0.05 and absolute fold change≥2 were considered differentially expressed genes/transcripts.

### Functional enrichment analysis

To identify and compare the overrepresented biological functions, enrichment analysis was performed using a hypergeometric test with our in-house R analysis package richR (http://github.com/hurlab/richR). Kyoto Encyclopedia of Genes and Genomes (KEGG) pathways, Gene Ontology (GO) terms were used in the enrichment analysis, and the calculated p-value were gone through FDR Correction, taking FDR ≤ 0.05 as a threshold. GO terms meeting this condition were defined as significantly enriched GO terms in DEGs. KEGG is the major public pathway-related database [45, 46]. Pathway enrichment analysis identified significantly enriched metabolic pathways or signal transduction pathways in DEGs comparing with the whole genome background.

### Methylation level analysis

The obtained clean reads were mapped to the species reference genome using BSMAP software (version: 2.90) [47]. Then a custom Perl script was used to call methylated cytosines and the methylated cytosines were tested with the correction algorithm described in Lister R. et al. (2009) [48]. The methylation level was calculated from the percentage of methylated cytosines per sequence context (CG, CHG and CHH) across the whole genome, each chromosome and different regions of the genome. To assess different methylation patterns in different genomic regions, the methylation profile of the flanking 2 kb regions and gene body (or transposable elements) was plotted based on the average methylation levels for each window.

### Differentially methylated cytosines (DMCs) and differentially methylated regions (DMRs) analysis

Differential DNA methylation between the two groups at each locus was determined using Pearson’s chi-square test (χ2) in methyl Kit (version: 1.7.10) [49]. To identify differentially methylated cytosines (DMCs), the minimum read coverage to call a methylation status for a base was set to 4. Differentially methylated cytosines for each sequence context (CG, CHG and CHH) between two groups were identified according to different criteria. And the differentially methylated regions (DMRs) also were identified according to different criteria.

### Consent for publication

All authors consent to publish the work.

### Availability of data and materials

The whole-genome bisulfite sequencing (WGBS) and RNA sequencing (RNA-seq) data from this study have been deposited in the Genome Sequence Archive in BIG Data Center (http://bigd.big.ac.cn/), Chinese Academy of Sciences, under the accession number: HRA002227; HRA002236.

## Supplementary Material

Supplementary Tables
