# Supplementary material for: Genome-wide methylation and gene-expression analyses in thalassemia
Source: Aging (Albany NY). 2024 Aug 9;16(15):11591–605. doi: 10.18632/aging.206037 (PMC11346785; doi:10.18632/aging.206037)
Supplement: Supplementary Tables [file aging-16-206037-s001.pdf]

SUPPLEMENTARY TABLES

Supplementary Table 1. Table of transcriptome sequencing raw data filtering statistics.

Sample	RawDatas	CleanData(%)	Adapter(%)	LowQuality(%)	polyA(%)	N(%)
Th1	50006004	49902462 (99.79%)	18724 (0.04%)	84640 (0.17%)	0 (0.00%)	178 (0.00%)
Th2	55443846	55318884 (99.77%)	18758 (0.03%)	106046 (0.19%)	0 (0.00%)	158 (0.00%)
Th3	40425602	40328798 (99.76%)	14374 (0.04%)	82310 (0.20%)	0 (0.00%)	120 (0.00%)
Th4	50783392	50666806 (99.77%)	15442 (0.03%)	100984 (0.20%)	0 (0.00%)	160 (0.00%)
Th6	41425714	41330790 (99.77%)	11046 (0.03%)	83752 (0.20%)	0 (0.00%)	126 (0.00%)
Th7	48321188	48208000 (99.77%)	17604 (0.04%)	93980 (0.19%)	0 (0.00%)	1604 (0.00%)
Th8	49510754	49381966 (99.74%)	19880 (0.04%)	107278 (0.22%)	0 (0.00%)	1630 (0.00%)
Th9	41641166	41549312 (99.78%)	17202 (0.04%)	72980 (0.18%)	0 (0.00%)	1672 (0.00%)
Th10	50783392	50666806 (99.77%)	15442 (0.03%)	100984 (0.20%)	0 (0.00%)	160 (0.00%)
Th11	42764640	42666194 (99.77%)	16834 (0.04%)	79844 (0.19%)	0 (0.00%)	1768 (0.00%)
Th12	49234158	49125144 (99.78%)	18574 (0.04%)	88456 (0.18%)	0 (0.00%)	1984 (0.00%)
Th13	52239378	52129862 (99.79%)	14880 (0.03%)	93242 (0.18%)	0 (0.00%)	1394 (0.00%)
Th14	46151816	46051722 (99.78%)	14316 (0.03%)	84262 (0.18%)	0 (0.00%)	1516 (0.00%)
N2	44249462	44147832 (99.77%)	22396 (0.05%)	77442 (0.18%)	0 (0.00%)	1792 (0.00%)
N3	40172616	40101378 (99.82%)	11700 (0.03%)	57826 (0.14%)	0 (0.00%)	1712 (0.00%)
N4	47446602	47361862 (99.82%)	11574 (0.02%)	71458 (0.15%)	0 (0.00%)	1708 (0.00%)
N5	47509608	47425176 (99.82%)	12592 (0.03%)	70254 (0.15%)	0 (0.00%)	1586 (0.00%)
N6	47154318	47062864 (99.81%)	15354 (0.03%)	74538 (0.16%)	0 (0.00%)	1562 (0.00%)
N7	40852226	40773584 (99.81%)	13550 (0.03%)	63426 (0.16%)	0 (0.00%)	1666 (0.00%)
N8	53202306	53087908 (99.78%)	16950 (0.03%)	95996 (0.18%)	0 (0.00%)	1452 (0.00%)

Supplementary Table 2. Table of transcriptome data comparison reference statistics table.

Sample	Total	Unmapped(%)	Unique_Mapped(%)	Multiple_Mapped(%)	Total_Mapped(%)
Th1	49316178	1192760 (2.42%)	46786713 (94.87%)	1336705 (2.71%)	48123418 (97.58%)
Th2	54484068	1539106 (2.82%)	51421781 (94.38%)	1523181 (2.80%)	52944962 (97.18%)
Th3	39512696	1134702 (2.87%)	37355273 (94.54%)	1022721 (2.59%)	38377994 (97.13%)
Th4	49564172	1434264 (2.89%)	45467523 (91.73%)	2662385 (5.37%)	48129908 (97.11%)
Th6	39645906	1080551 (2.73%)	37507972 (94.61%)	1057383 (2.67%)	38565355 (97.27%)
Th7	47612628	1366704 (2.87%)	44619278 (93.71%)	1626646 (3.42%)	46245924 (97.13%)
Th8	48803368	1467442 (3.01%)	46079180 (94.42%)	1256746 (2.58%)	47335926 (96.99%)
Th9	41023672	1122839 (2.74%)	38860892 (94.73%)	1039941 (2.53%)	39900833 (97.26%)
Th10	48552004	1397544 (2.88%)	45777749 (94.29%)	1376711 (2.84%)	47154460 (97.12%)
Th11	42289604	1276988 (3.02%)	39704597 (93.89%)	1308019 (3.09%)	41012616 (96.98%)
Th12	47466072	1457269 (3.07%)	44642002 (94.05%)	1366801 (2.88%)	46008803 (96.93%)
Th13	50973230	1472993 (2.89%)	47266662 (92.73%)	2233575 (4.38%)	49500237 (97.11%)
Th14	44509112	1179953 (2.65%)	41900120 (94.14%)	1429039 (3.21%)	43329159 (97.35%)
N2	43472084	1180433 (2.72%)	41033506 (94.39%)	1258145 (2.89%)	42291651 (97.28%)
N3	39785296	1022730 (2.57%)	37570857 (94.43%)	1191709 (3.00%)	38762566 (97.43%)
N4	46871142	1163005 (2.48%)	44295823 (94.51%)	1412314 (3.01%)	45708137 (97.52%)
N5	46406672	1292575 (2.79%)	43923147 (94.65%)	1190950 (2.57%)	45114097 (97.21%)
N6	46543924	1191423 (2.56%)	43894398 (94.31%)	1458103 (3.13%)	45352501 (97.44%)
N7	40434784	1040819 (2.57%)	38290611 (94.70%)	1103354 (2.73%)	39393965 (97.43%)
N8	52532404	1543542 (2.94%)	49433409 (94.10%)	1555453 (2.96%)	50988862 (97.06%)

Supplementary Table 3. Table of methylation data filter.

Sample	Clean reads num	HQ Clean reads num (%)
Th1	655011516	643133678 (98.19%)
Th2	578756378	571110174 (98.68%)
Th3	591190230	581830674 (98.42%)
Th4	633080368	621180388 (98.12%)
Th5	623730348	620351826 (99.46%)
Th6	633309176	618384592 (97.64%)
Th7	611371898	597365680 (97.71%)
Th8	602652242	589629610 (97.84%)
Th9	620137130	609657726 (98.31%)
Th10	617834478	613911016 (99.36%)
Th11	589514406	580554920 (98.48%)
Th12	597078888	586488056 (98.23%)
Th13	594013150	587207694 (98.85%)
N1	590001510	583815932 (98.95%)
N2	653835408	641331770 (98.09%)
N3	586415144	578223570 (98.6%)
N4	581657012	573956604 (98.68%)
N5	621917978	615017628 (98.89%)
N6	648774992	636028996 (98.04%)
N7	640678680	627502560 (97.94%)
N8	583698310	575715568 (98.63%)
